# Play ontogeny in young chickens is affected by domestication and early stress

**DOI:** 10.1038/s41598-022-17617-x

**Published:** 2022-08-09

**Authors:** Lundén Gabrielle, Oscarsson Rebecca, Hedlund Louise, Gjøen Johanna, Jensen Per

**Affiliations:** IFM Biology, AVIAN Behaviour Genomics and Physiology Group, Linköping University, 581 83 Linköping, Sweden

**Keywords:** Animal behaviour, Zoology

## Abstract

Play is common in young homeotherm animals and has an important role as a tentative indicator of positive states of welfare. Furthermore, during domestication play is believed to have increased in frequency in several species as part of the domestication syndrome. Here, we studied the ontogeny of play in chickens in two experiments. The first compared the behavioural development between domesticated White Leghorn (WL) laying hen chicks and ancestral Red Junglefowl (RJF) and the second compared the same between WL chicks that had experienced the stress of commercial hatchery routines and a control group, hatched under calm conditions. In both experiments, 10 groups of four chicks each from each of the groups were moved twice per week to an enriched and fully enclosed play arena, starting at day 8 and finishing day 39 or 53 after hatch. In the arena, the frequency of play behaviours was recorded during 30 min and divided into object, locomotory and social play. In experiment one, total play as well as object play was significantly more common in WL whereas locomotor and social play was more common in RJF. In experiment two, total play was significantly more frequent in commercially hatched chicks, despite that none of the sub-categories differed significantly between the groups. In conclusion, domestication as well as early stress does affect the occurrence of play in chickens, but the effects are complex and require further research.

## Introduction

Play is ubiquitous in young homeothermic animals, including many birds^1^. Despite its common occurrence, play is surprisingly difficult to define in a way that reliably allows observers to distinguish it from non-playful behaviour. This is not least the case in chickens and is probably one reason for the scarcity of play studies in this species^2^. In an attempt to provide a general classification scheme, it has been suggested that actions classified as play should meet five different criteria^3^: (1) it consists elements that do not contribute towards the animal’s immediate survival; (2) it is spontaneous or rewarding in itself; (3) it differs from the functional variant of the behaviour either in structure or temporal organization; (4) it is repeated in similar forms throughout at least parts of the ontogeny; (5) it primarily occurs when the animal is in a state of good health and free from stress. Furthermore, it has been implicated that play behaviour is intrinsically characterized by positive emotions^4^. These aspects mean that play behaviour can potentially serve as an indicator of positive welfare states^5^. Studies of this kind have been performed on, for example, lambs^6^, calves^7^, and pigs^8^.

Play behaviour occurs in different categories and can be broadly divided into object play (involving manipulation of different items), locomotor play (e.g., running, jumping, frolicking), and social play (e.g., sparring, wrestling)^1^. Play in young chickens were described by Kruijt in his account of the ontogeny of social behaviour in Red Junglefowl^9^ and some differences between chicken breeds in the occurrence of play behaviour such as running and frolicking have been observed, suggesting a genetic basis for variation in play^10^. Only a few studies have systematically analysed play in young chickens as a means to assess their welfare, and these studies have mainly concerned fast-growing broilers. For example, it was found that, contrary to expectations, providing broiler chicks with extensive environmental enrichment reduced the occurrence of various play activities when compared to chicks in non-enriched pens^2^, and in a commercial broiler house, enrichment did not affect play in any significant way^11^. Hence, there is a need for fundamental studies of factors affecting the ontogeny of play in young chickens from different perspectives. In the present study we focus on the effects of domestication and of early stressful experiences.

Chickens were domesticated from ancestral Red Junglefowl about 8–9000 years ago^12,13^ and are now the most common terrestrial food-producing animals in the world^14^. In general, domestication of a species is associated with a range of modifications in appearance, physiology and behaviour, commonly known as the domestication syndrome^15^. This includes reduced fearfulness towards humans, reduced stress susceptibility and a prolonged retainment of juvenile traits^16^. Such retainment can also affect play behaviour, and for example domesticated guinea pigs play more than their wild ancestors^17^. In chickens, comparative studies have revealed a lack of qualitative differences in behaviour between the ancestor and modern domesticates^18^, although many behaviours have changed in a quantitative way^19^. Hence, we hypothesized that there would not be any qualitative differences in the play behaviour of Red Junglefowl and domesticated egg layers, but a possible increase in the frequency of play in the domesticates.

Stressful experiences early in life can cause long-time alterations in the welfare of chickens^20^. In present-day commercial egg production, chicks are hatched in large-scale commercial hatcheries, and this is associated with a range of stressful events during the first day of life^21^. The incubators and hatchers are noisy, and the chicks are processed on conveyor belts for manual sex sorting, vaccination, loading and finally several hours of road-transport to the rearing farms. We have previously shown that hatchery processing is associated with a significant short-time increase in plasma corticosterone levels^21^, and long-time effects on mood and welfare as shown by, e.g., a more “pessimistic” cognitive judgement bias than in chicks hatched under calm conditions^22^. Commercial hatching may therefore serve as a realistic model for early stress. Given that play is considered to be associated with states of good welfare, we hypothesized that commercially hatched chicks would show a reduced frequency of play throughout ontogeny.

The aim of this study was three-fold: (1) to characterize the ontogeny of different types of play behaviour in young chickens; (2) to describe any differences in play behaviour between ancestral and domesticated young chickens; (3) to assess any effects of early stress encountered during commercial hatching on play behaviour.

## Materials and methods

### Animals

#### Experiment 1

The aim of experiment 1 was to describe differences in the ontogeny of play between domesticated chickens and ancestral Red Junglefowl. The Red Junglefowl (RJF) chicks (n = 40) used in experiment 1 were from an unselected parental line of birds, bred and housed in the facilities at Linköping University (for detailed information about the background of these birds, see Schütz and Jensen^23^). The domesticated birds were White Leghorn (WL) chicks (n = 40) of the commercial egg-laying hybrid Lohmann LSL-LITE. RJF-eggs were collected from the home pens of the RJF during a period of 1 week, and WL-eggs were obtained from a commercial hatchery in Sweden 3 days prior to start of incubation. All eggs were incubated and hatched at Linköping University in the same small incubator set to 38.5 °C, 65% relative humidity and rotation once per hour. All chicks were incubated and hatched in darkness, and were separated in different common hatching trays by breed.

#### Experiment 2

For experiment 2, aiming at analysing any possible effects of early stress on the ontogeny of play, we used the WL-chicks from experiment 1 as control chicks, representing birds that were incubated and hatched under calm conditions. In addition, we obtained 40 newly hatched WL chicks from the same commercial hatchery. These chicks were from the same parental stock as the control chicks, and were incubated, hatched and taken out of the incubator at the corresponding time-point as the RJF and the control WL. Furthermore, the chicks from the commercial hatchery were handled in accordance with commercial routines, including conveying, sex sorting, vaccination, machine packing and transportation for 4 h (for more details about the commercial hatchery procedures, see Hedlund et al.^21^. After arrival at the university, they were kept under identical conditions as the control chicks throughout the experimental time. Hence, the experiment consisted of one group of chicks that had undergone the stress of commercial hatching and a matched control group of the same age and background that had not experienced early stress.

### Housing

All birds from both experiments were initially housed in sex-mixed groups of 20 individuals each (in total 6 groups, each in one pen), with early stressed WL, control WL and RJF each kept separate. The pens consisted of solid floor cages (W × L × H: 0.7 × 0.68 × 0.57 m) and were provided with wood chips, a heat roof, as well as feed and water ad lib.

At 23 days of age, all birds were moved to larger pens measuring 0.7 × 2.1 m, supplied with wood chips, a heat lamp, perches, feed, and water. From this point on, all birds within each category (WL control, WL early stress, RJF) were housed together (in total 3 groups with 40 birds in each). The pens were further expanded to 0.7 × 2.8 m on day 42 to accommodate for the growth of the birds. The animals were kept on a 12:12 h dark:light schedule throughout the experiment.

### Experimental set-up and procedure

After hatch, as the chicks were placed in their home pens, we created random groups of four birds, and each group was marked with unique codes of coloured leg rings. Each such group constituted the test unit and were always tested together throughout the experiment. The sample size was determined based on previous experiences of reasonable samples in behavioural studies of young chicks. Since this is, to our knowledge, the first study to provide quantitative assessments of play ontogeny in chickens, we had no a priori expectations of possible differences between breeds or groups, and could therefore not perform any a priori power analysis.

In both experiments, the chicks were tested at the following days of age: 8, 10, 15, 18, 22, 25, 29, 32, 36 and 39. Birds in experiment 1 were additionally tested at days 43, 46, 50 and 53. Due to the fact that it is not possible to differ between males and females in young Red Junglefowl chicks, and very difficult in White Leghorns, we were not able to break down the analyses on sex. Each group therefore had a random sex composition.

It has been shown that play in chicks is stimulated by increased access to space and objects to play with^2^. Therefore, the tests were carried out in separate, fully enclosed arenas that were considerably larger than the home pens (L × W × H: 1.17 × 0.8 × 1 m), containing wood chips, a perch along one short end, a small pile of hay, and a hanging chain. As the arenas were completely enclosed, there were no visual stimuli from outside the arena. Up to day 23, the home pens were situated in the same lab as the test arenas, 3–5 m away from those, and thereafter the home pens were in an adjacent building, 3 min walking distance from the lab. For each test session, the chicks were gently caught and moved in a fully enclosed transport box from their home pens and placed in the arena with lights off. Chicks normally remain calm and still in darkness, and the procedure helped keeping stress due to moving at a low level. After 1–2 min, the light was turned on and the recordings started. This allowed us to start all test sessions simultaneously in all four parallel arenas. There were four identical parallel arenas in the lab, allowing simultaneous testing of four groups.

Each test session lasted for 30 min and during this time the behaviour was recorded through overhead video cameras. Ten minutes into the test, a fake worm made of rubber was presented to the birds, via a small opening with a lid in one corner of the arena, ensuring that the birds did not see the person entering the object. During the first two test days, a fake worm measuring 2 × 60 mm was used, and this was then replaced with a larger one (3 × 165 mm) that was used throughout the rest of the testing period. After an additional 10 min, a small cardboard box with three live mealworms were inserted in the arena through a similar small opening in the opposite corner.

From the videos, the occurrence of 14 different play behaviours was scored, and these were subsequently grouped into three larger categories: locomotor play, social play and object play. Furthermore, all occurrences of any behaviour were summed into one category called “Total play”. Locomotor play included running, frolicking, wing flapping, spinning, and spinning while wing flapping. Object play included object running, worm running, object/worm chasing, object/worm exchange and worm pecking. Social play included sparring jumping with or without contact, sparring stand-off with or without contact.

A complete ethogram with detailed descriptions of all included behaviours is provided in Supplementary Table S1. The complete data set for both experiments is provided in Supplementary Table S2. Video footage showing examples of the different play behaviours is provided in Supplementary Videos S3, S4, and S5.

### Sampling and data analysis

The occurrence of the different behaviours were recorded in 15 s segments throughout the 30 min. During each time segment, we used 1/0 sampling for each chick, i.e., for every segment of 15 s, we recorded how many of the four individuals that performed each behaviour at least once. Hence, in every segment the recorded frequency of each behaviour could vary from 0 to 4, and for the entire 30 min test, the total number of observations of each behaviour could vary from 0 (meaning that the behaviour was never observed) to 480 (meaning that all four birds performed the behaviour at least once during all 15 s segments).

The videos were scored by two observers, where one scored experiment 1 and the other experiment 2. To assess the validity of the scoring, 25% of the videos were scored by both observers independently and we calculated the Spearman correlation coefficients as a measure of inter-observer agreement. The correlations were: locomotor play: 0.89; social play: 0.91; object play: 0.97; total play: 0.93. All correlations were significant (P < 0.001).

Every group of four birds constituted the independent statistical replicates. We used Generalized Linear Mixed Models with a repeated measures design to analyse the effects of age, breed and their interactions in experiment 1, and age, hatchery treatment and their interactions in experiment 2. When interactions were not significant, they were removed and the model re-run with only the main factors. The model was fitted for negative binomial distribution with log-link function since the data were made up of counts with a high variance. All data are presented as average number of observations per group with standard error of the mean. The statistical analyses were performed in SPSS 28.0.1.

### Ethical approval

All experimental protocols were approved by Linköping Council for Ethical Licensing of Animal Experiments, ethical permit no 14916-2018. Experiments were conducted in accordance with the ARRIVE guidelines. The protocol was performed in accordance with the relevant guidelines and regulations.

## Results

### Experiment 1: Domestication effects

**Figure 1:**
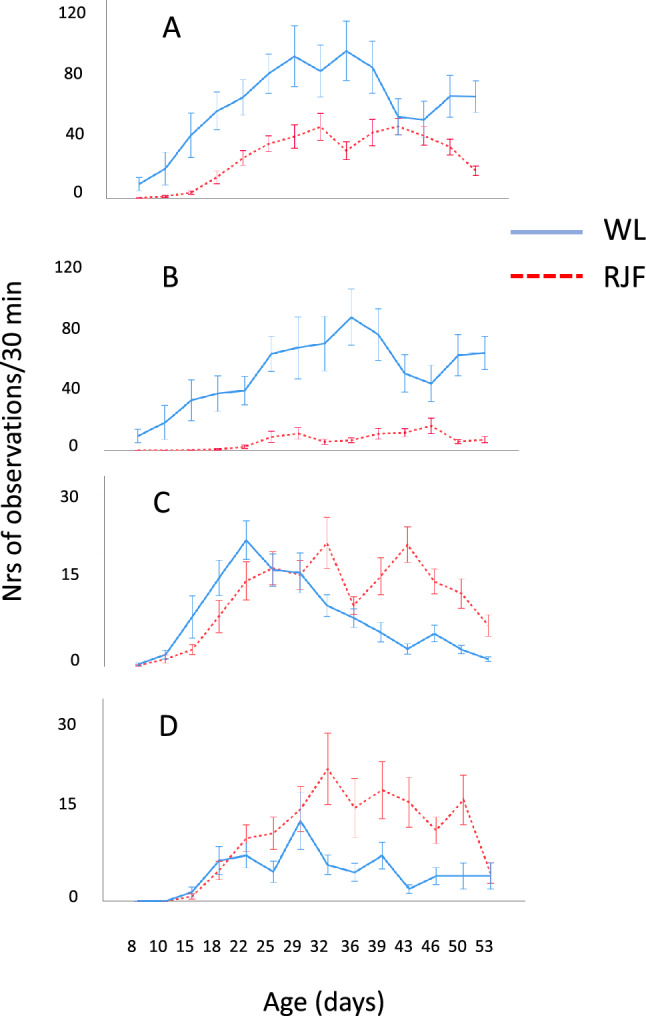
Mean number of observations (± standard error) per group per 30 min of (**A**) total play, (B) object play, (**C**) locomotor play, and (**D**) social play at different ages in Red Junglefowl and domesticated White Leghorn chicks. Note that the scales differ between the graphs.

There were no qualitative differences observed between the Red Junglefowl (RJF) and the domesticated White Leghorns (WL). All 14 behavioural categories were recorded in both breeds, and there were no apparent differences in how they were performed. There was a significant interaction between breed and age (Fig. 1A; F_13, 252_ = 3.5, P < 0.001), caused by the fact that total play peaked earlier in WL. The frequency of total play (all play behaviours summed) showed a significant change with age in both breeds, with very few occurrences in the first 2 weeks of age followed by an increasing frequency until a peak between 25 and 36 days in WL and 32–43 days in RJF, further followed by a gradual decrease thereafter (Fig. 1A; effects of age: F_13, 252_ = 10.0, P < 0.001). Domesticated WL performed significantly more total play behaviour compared to the RJF (Fig. 1A; F_1, 252_ = 106.1, P < 0.001).

The most common of the subtypes of play was object play (Fig. 1B), whereas both locomotor and social play occurred at considerably lower frequencies (Fig. 1C,D). Object play was more frequent in WL (F_1, 252_ = 5.9, P < 0.05), whereas both locomotor and social play were more frequent in RJF (locomotor play: F_1, 252_ = 8.9, P < 0.01; social play: F_1, 252_ = 25.4, P < 0.001).

All three subtypes followed similar ontogenetic patterns as for total play, with peaks occurring earlier in WL than in RJF. All subtypes showed significant age effects (object play: F_13, 252_ = 2.7, P < 0.001; locomotor play: F_13, 252_ = 15.9, P < 0.001; social play: F_13, 252_ = 265.4, P < 0.001). Furthermore, there were significant interactions between type of play and age, confirming that the different play types peaked at different ages in the two breeds (object play: F_13, 252_ = 2.7; P < 0.01; locomotor play: F_13, 252_ = 6.5, P < 0.001; social play: F_13, 252_ = 3.8, P < 0.001).

### Experiment 2: Effects of early stress

**Figure 2:**
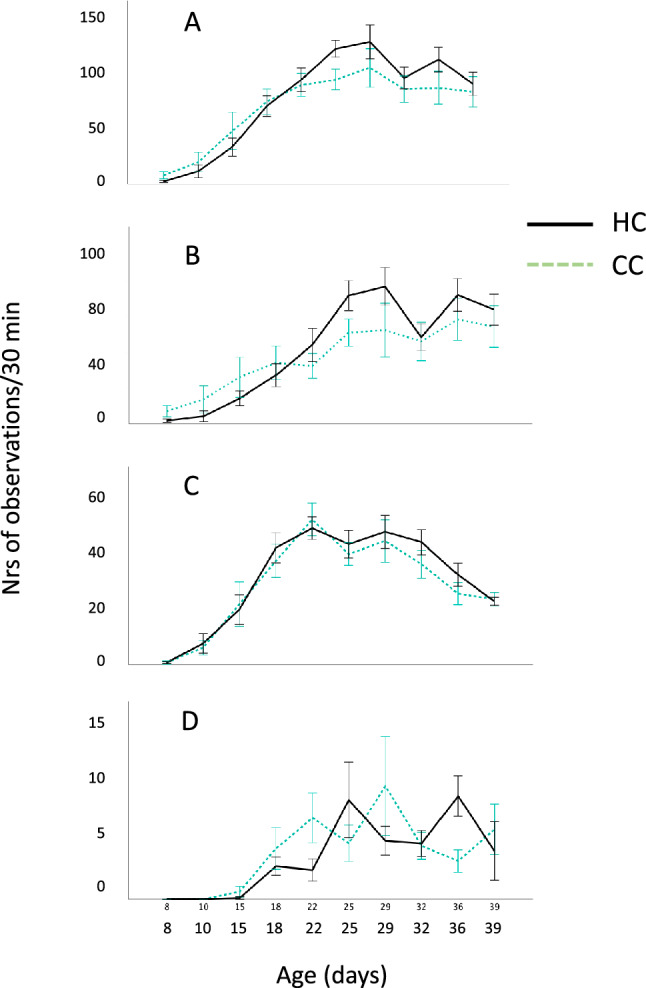
Mean number of observations (± standard error) per group per 30 min of (**A**) total play, (**B**) object play, (**C**) locomotor play, and (**D**) social play at different ages in White Leghorn chicks hatched in a commercial hatchery (HC) or under calm conditions (CC). Note that the scales differ between the graphs.

There was no significant interaction between age and treatment for total play (F_1, 180_ = 0.32, P = 0.9). However, the frequency of total play (all play behaviours summed) again showed a significant change with age, both groups following a similar ontogenetic pattern as shown in experiment 1 (Fig. 2A; effects of age: F_13, 189_ = 13.8, P < 0.001). Hatchery chicks played significantly more than control chicks, although the numerical differences were relatively small compared to the breed differences in experiment 1 (Fig. 2A; F_1, 189_ = 4.4, P = 0.037).

As in experiment 1, object play was the most common of the subtypes (Fig. 2B), with locomotor and in particular social play occurring at a much lower frequency (Fig. 2C,D). All three subtypes showed significant age effects (object play: F_9, 189_ = 8.8, P < 0.001; locomotor play: F_9, 189_ = 21.7, P < 0.001; social play: F_9, 180_ = 210.9, P < 0.01). However, there were no significant differences between the treatment groups in any of the three subtypes of play when analysed separately, although there was a tendency for more object and social play in the hatchery chicks (object play: F_1,189_ = 3.2, P = 0.075; locomotor play: F_1,189_ = 0.9, P = 0.34; social play: F_1, 180_ = 3.2; P = 0.073). There was a significant interaction between age and treatment for social play (F_9, 180_ = 2.7, P < 0.01), but not for any of the other subtypes (object play: F_9, 180_ = 1.3, P = 0.22; locomotor play: F_9, 180_ = 0.3, P = 0.9).

## Discussion

We found no qualitative differences in play behaviour between ancestral Red Junglefowl (RJF) and domesticated White Leghorn (WL) chicks, but WL chicks had significantly more total play, mainly caused by more object play, while RJF performed more of the less frequent locomotory and social play. The ontogenetic development of play followed similar patterns in both breeds but peaked earlier in WL. In WL laying hen chicks, early stress from commercial hatching was associated with a small but significant increase in the frequency of play. Hence, our results suggest that play behaviour in young chickens is affected by both domestication and early stress, but the effects are complex and require further research.

Studies of play in various species has shown its importance as welfare indicator. For example, lambs are highly motivated to obtain access to a pen that offers the possibility to play^6^, and painful disbudding reduces play behaviour in calves^7^. Despite the growing interest in play behaviour in different species, very little research has focused on chickens^2,5,24,25^.

One reason for the limited efforts to include chickens in this research might be the difficulties associated with distinguishing play from serious activities in this species. In a variety of species, play is most common during the early ontogeny, before and around nutritional independence from the parents^24^. Using this as a background, we developed a detailed ethogram based on previously published studies and own observations and divided the different behaviours into three categories: object, locomotor and social play. We then recorded the frequencies of the different activities over the juvenile period, repeatedly stimulating groups of chicks to play by giving them access to an enriched and enlarged arena, a procedure known to induce play in different species^5^. The fact that all three categories of behaviour showed a similar ontogenetic pattern, peaking at 30–40 days of age followed by a gradual decline as the chicks approached the age at which they would become independent in nature^14^, is interpreted as a strong indication that the different behaviours included in the ethogram were proper play activities. Among the three categories, object play was by far the most prevalent. Object play included worm running and worm pecking, both of which could of course also be interpreted as mainly feeding motivated behaviours. However, the fact that they both followed the same ontogentic pattern as the other activities in the ethogram strengthen the interpretation that both worm running and worm pecking are primarily play behaviours.

Domesticated animals share a set of phenotypic traits that differentiate them from their ancestors, such as reduced fearfulness and a modified ontogeny^15^. This is partly a result of similar selection pressures, including the necessity to thrive and reproduce in the proximity of humans^18^. To our knowledge, there have been no previous systematic comparisons of play behaviour in a domesticate and its wild ancestor, although some studies indicate that dogs play more and in a different way than wolves^26^ and it has been reported that guinea pigs play more than their wild counterparts^17^. Since one of the prominent traits of the domestication syndrome is intensification and prolongation of juvenile traits^15,16^, we hypothesized that domesticated chickens would play more than ancestral RJF. Whereas this was confirmed when considering the total frequency of play, the picture was slightly different when considering each different play category separately. Domesticated chickens played significantly more with the objects provided in the play arena, whereas the RJF engaged more in social and locomotory play. This is in agreement with the few reports existing on dog-wolf comparisons, where dogs do not only play more, but also engage in different types of play compared to wolves^26^. Hence, our results indicate that domestication may have caused chickens to become more playful, and that this playfulness is primarily directed towards objects. Furthermore, we found no differences in the types of play behaviours performed, showing that there have not been any qualitative behavioural modifications during domestication.

For all types of play, the frequency peaked earlier in WL. This may be related to the faster ontogenetic development in domesticated animals, a general aspect of the domestication syndrome^15^. For example, experimentally domesticated silver foxes open their eyes earlier and become sexually mature earlier than their non-domesticated conspecifics^16^. Domesticated chickens become sexually mature before 20 weeks of age compared to about 25 weeks in RJF^27^. Possibly, this increased speed of development could be a reason for the earlier play peak in WL.

The difference in the frequency of play between the breeds may partly be a result of differences in fearfulness. Previous studies have shown that RJF are more fearful than domesticated chickens in a range of different situations, despite being hatched and reared under identical conditions^28^. Hence, it is possible that the RJF chicks perceived the test arena and the objects therein, as well as being moved from their home pens, as more frightening than the WL, and this may have reduced their motivation to play.

In our second experiment, we studied the ontogeny of play behaviour in WL chicks with a history of early stress caused by commercial hatchery processing. We have previously shown that commercial hatching induces a long-term reduction in welfare, as observed, e.g., by a more pessimistic cognitive judgement bias maintained for several weeks after hatching^22^. We hypothesized that this negative state of mood would be associated with a reduced frequency of play compared to control chicks hatched under calm conditions, but in fact we observed the opposite. This was again mainly caused by a difference in object play, whereas there were negligible differences in locomotor and social play.

Our results concur with one of the few previous studies that have been published on play in chicks, in which it was found that broiler chicks raised in enriched pens played less than those in a more barren environment, contrary to the expectations^2^. The authors speculated that the unexpected results could have been due to the chicks from the barren environment experiencing a higher contrast in the play situation compared to their home pens, thus stimulating more play. Our results could possibly have a similar explanation, in that birds with a negative state of mood may have experienced a more intense stimulation when transferred to the play arena. Since we did not record the spontaneous play events in the home pens, it is still possible that early stressed chicks performed less of this, but this remains to be explored in future research. Another possibility is that the early stress experienced by commercially hatched chicks may in fact have made them more tolerant to later stress. Such “priming” effects of early stress have been demonstrated in a variety of species^29^, including chickens^30^. Hence, the increased play may possibly in fact reflect a more positive state of mood in the commercially hatched chicks. Of course, it also remains a possibility that play is not affected by stress and mood in chickens to the same extent as in other species, and as such would not be a suitable welfare indicator. More research is clearly needed in order to differentiate between the possible explanations.

It has been suggested that a way to improve the psychological welfare of farm animals could be to stimulate play and thereby offer them positive and rewarding experiences during ontogeny^5^. Our results clearly demonstrate that it is possible to stimulate different kinds of play in young chickens. An important route for future research would therefore be to study the long-time effects of such play stimulation on cognitive development and other welfare traits.

In conclusion, we found that play in chickens peaks between 25 and 40 days of age and is dominated by object play. Furthermore, although there were no qualitative differences in the types of play behaviour performed, domesticated chickens engaged more in play than ancestral Red Junglefowl, and early stress tended to increase the frequency of play in a stimulating play arena. Future studies should investigate the possibilities of improving the welfare of chickens in the egg production by stimulating play during early ontogeny.

## Supplementary Information

Supplementary Information 1.

Supplementary Information 2.

Supplementary Information 3.

Supplementary Video 1.

Supplementary Video 2.

Supplementary Video 3.

## Data Availability

The datasets generated and/or analysed during the current study are available in the supplementary information, Table S2. Questions regarding the data can be directed to the corresponding author, per.jensen@liu.se.
