# Supplementary material for: Play ontogeny in young chickens is affected by domestication and early stress
Source: Sci Rep. 2022 Aug 9;12:13576. doi: 10.1038/s41598-022-17617-x (PMC9363459; doi:10.1038/s41598-022-17617-x)
Supplement: Supplementary file 3 — Supplementary Information 3. [file 41598_2022_17617_MOESM3_ESM.docx]

**Play ontogeny in young chickens is affected by domestication and early stress**

Lundén, Gabrielle; Oscarsson, Rebecca; Hedlund, Louise; Gjøen, Johanna; Jensen, Per

**Supplementary information: Labeling of supplementary videos S3, S4, and S5**

**Supplementary video S3:** Examples of Locomotor play behaviour. The video shows running, frolicking, wing flapping, spinning while wing flapping.

**Supplementary video S4:** Examples of social play behaviour. The video shows Sparring jumping with no contact, sparring jumping with contact, sparring stand-off with no contact, and sparring stand-off with contact.

**Supplementary video S5:** Examples of Object play behaviour. The video shows worm running (both with meal worm and rubber worm), worm chasing, and worm exchange.
